# Characterization of globulin storage proteins of a low prolamin cereal species in relation to celiac disease

**DOI:** 10.1038/srep39876

**Published:** 2017-01-04

**Authors:** Gyöngyvér Gell, Krisztina Kovács, Gábor Veres, Ilma R. Korponay-Szabó, Angéla Juhász

**Affiliations:** 1 Agricultural Institute, MTA Centre for Agricultural Research, Department of Applied Genomics Martonvásár, HU 2462, Hungary; 2 Semmelweis University of Medicine, 1st Department of Pediatrics, Budapest, HU 1083, Hungary; 3 Coeliac Disease Center, Heim Pál Children’s Hospital, Budapest, HU 1089 and Department of Pediatrics, Clinical Center, University of Debrecen, Debrecen, Hungary

## Abstract

*Brachypodium distachyon,* a small annual grass with seed storage globulins as primary protein reserves was used in our study to analyse the toxic nature of non-prolamin seed storage proteins related to celiac disease. The main storage proteins of *B. distachyon* are the 7S globulin type proteins and the 11S, 12S seed storage globulins similar to oat and rice. Immunoblot analyses using serum samples from celiac disease patients were carried out followed by the identification of immune-responsive proteins using mass spectrometry. Serum samples from celiac patients on a gluten-free diet, from patients with Crohn’s disease and healthy subjects, were used as controls. The identified proteins with intense serum-IgA reactivity belong to the 7S and 11–12S seed globulin family. Structure prediction and epitope predictions analyses confirmed the presence of celiac disease-related linear B cell epitope homologs and the presence of peptide regions with strong HLA-DQ8 and DQ2 binding capabilities. These results highlight that both MHC-II presentation and B cell response may be developed not only to prolamins but also to seed storage globulins. This is the first study of the non-prolamin type seed storage proteins of *Brachypodium* from the aspect of the celiac disease.

Immune reactive peptides of prolamin type storage proteins of cereal species such as wheat, rye and barley are considered as trigger molecules in the development of celiac disease (CD), a systemic autoimmune disease causing villous atrophy in the small intestine. Celiac disease has a strong genetic component and occurs only in subjects carrying the human leukocyte antigen (HLA) DQ2.5, DQ8 or DQ2.2 haplotypes which act as cell surface receptors for the toxic peptides on antigen presenting cell. Deamidation of wheat gliadin peptides by gastric acid or by certain enzymes (e.g. the main celiac autoantigen, type-2 transglutaminase), strongly increases their immunogenic properties by making them properly fitting into the binding groove of DQ2 and DQ8. Antibodies against these deamidated gliadin peptide (DGP) antibodies have higher diagnostic value than conventional anti-gliadin (AGA) antibodies^1^,^2^. Today the only effective treatment is a lifelong gluten-free diet that is often rich in rice, corn, quinoa, teff or amaranth. The common characteristic of these whole grain sources is that their seed is rich in storage globulins instead of prolamin like storage proteins.

*Brachypodium distachyon* is a diploid annual wild grass that belongs to the *Pooideae* subfamily of the grasses, and based on the recent phylogenetic analyses is the closest wild relative of wheat and barley^3^,^4^. However, its storage protein composition and the extraction behaviour of these proteins make *B. distachyon* closer to rice or oat than to wheat endosperm proteins^5^. *B. distachyon* accession Bd21 offers many advantages, such as self-fertility, simple nutrient requirements and short lifecycle. Sequencing and annotation of the Bd21 genome were recently completed, making further functional proteomic studies feasible^6^,^7^,^8^. Along with previous results of Laudencia-Chingcuanco and Vensel^9^, the study by Larré and co-workers^5^ has revealed that the main storage proteins of *B. distachyon* are the 11S, 12S and 7S globulin type proteins similar to oat and rice. The prolamins, including the avenin-like proteins and the gliadin-like prolamins, represent less than 12% from the total protein content that is significantly lower compared to wheat. 11S–12S globulins account for 70–80% of total seed protein content^5^. Due to its low prolamin content, *B. distachyon* may serve as an excellent source to investigate the immune-reactive capacity of non-prolamin seed proteins. In our previous study, the published chromosome specific *B. distachyon* genome sequences and a seed specific cDNA library data were used for sequence-based identification of proteins with regions identical to known celiac disease-specific epitopes^10^. Next to proteins with nutrient reservoir activity proteins with nucleic acid or protein binding activities, transcription regulator function and catalytic functions were identified. Although the percentage of epitope containing proteins with nutrient reservoir activity was low (6.8%), this value has increased when epitope homologues over 80% identity were also considered. These results have highlighted the presence of possible cross-reactive epitope homologues to celiac disease related trigger molecules. Although *Brachypodium* is not considered for human nutrition, we took advantage of its use as a model species for the understanding whether abundant non-prolamin cereal seed proteins with linear epitope homologues play a role in the development of humoral immune response and to help select other food sources suitable for gluten free diet. In our study high-resolution proteomic analysis of total protein extracts of mature *Brachypodium* seeds and serum samples of patients suffering in celiac disease were used to characterize seed proteins with elevated immune reactivity.

## Materials and Methods

### Patients and Sera

Serum samples from celiac patients with known HLA-DQ haplotypes and positive for celiac disease antibodies on gluten intake (n = 13, 8 females, 5 males), median age 5.7 years, range 1.4–13.5 years).Serum samples from celiac patients adhering to a strict gluten-free diet (GFD) resulting in normalised antibodies (anti-transglutaminase IgA < 10 U/l) and mucosal healing (n = 3), from ten newly diagnosed Crohn’s disease patients with ileocolon manifestation (median age 6.4) and eight healthy control subjects were used for immunoblotting studies. Celiac disease was diagnosed according to the criteria of ESPGHAN (European Society of Paediatric Gastroenterology, Hepatology and Nutrition) and according to Marsh criteria and all patients had Marsh III grade intestinal lesions and elevated IgA anti-transglutaminase (TG2) IgA and IgG antibody serum levels (>200 U/l)^11^,^12^. Six celiac patients were HLA DQ2.5 homozygous; five were DQ8 homozygous, and one was heterozygous (DQ8/X). The exact DQ typing result was not available in one other case. One of the DQ8 cases developed type-1 diabetes mellitus two years after celiac disease diagnosis. Informed written consent to the collection of serum samples was given by the parents. All the methods of subject recruitment, data collection, and experiments were performed in accordance with relevant guidelines and regulations. All experiments were approved by the ethics committee of the Heim Pál Children’s Hospital, Budapest and the Semmelweis University Regional and Institutional Committee and Research Ethics. Serum samples were collected during the diagnostic procedure of celiac disease for the clinical investigation of celiac antibodies according to ESPGHAN guidelines and used later for the experiments with the permission of the Ethical Committee of the Heim Pal Children’s Hospital, Budapest.

### Protein Extractions

Total protein of 50 mg-s of crushed *B. distachyon* inbred line Bd21 seeds were extracted following the protocol of Dupont and co-workers^13^. Briefly, an SDS-Tris extraction buffer (pH 6.8) containing 2% SDS, 10% glycerol, 50 mM DTT and 40 mM Tris-HCl, pH 6.8 was used to extract the total protein at room temperature for one hour with a regular gentle vortexing. The extract was centrifuged at 16000 g for 15 minutes. The supernatant was precipitated with four volume ice-cold acetone. Five biological replicates were used in the extraction process.

### 2D Gel Electrophoresis and Immunoblotting

Following the precipitation the pellet was solubilized with IEF buffer (8 M urea, 2% CHAPS, 100 mM DTT, 0.2% CA and 0.1% Bromophenol Blue). The isoelectric focusing was carried out in 7 cm Immobiline DryStrips pH 3–10 (GE Healthcare), under overnight rehydrating conditions with 200 μg protein in 150 μl IEF buffer. 12% polyacrylamide gels were used to separate proteins based on their molecular weight. 2D GE was made in three technical replicates. Because all of the 2D protein patterns from the different biological and technical replicates were identical, the final on-line nano-LC-MS/MS was made from three technical replicates 2D GE, and the spots were bulked.

After the 2D GE proteins were transferred to ImmobilonP PVDF membrane (Millipore, Billerica, USA) and blocked for 1 hour with 5% BSA and 0.05% TWEEN20 followed by an overnight incubation at 4 °C with 1:20 diluted blood sera. The immune-reactive proteins were detected with anti-Human IgA peroxidase- conjugated antibody produced in goat (Sigma-Aldrich-A0295) in the presence of 4-Chloro-1-Naphthol chromogenic peroxidase substrate. Immune responsive protein spots were excised and sent for protein identification using on-line nano-LC-MS/MS. Rice glutelin antibody coupled with anti-rabbit IgA as a secondary antibody was used in 2D Western blot analysis to confirm the presence of seed storage globulins^14^.

### Protein Identification by on-line nano-LC-MS/MS

Gel spots were in-gel digested for 4 hrs at 37 °C with sequencing grade modified Trypsin (Promega, V5111) after reduction with DTT (Sigma-Aldrich) and alkylation with Iodoacetamide (Sigma-Aldrich). The peptide extracts were analysed by mass spectrometry, using on-line nano-LC-MSMS technique on a Waters nano-Acquity UPLC coupled with Thermo LTQ-Orbitrap-Elite mass spectrometer. Ion-trap CID spectra were acquired from the ten most intense peaks after each survey scan. Proteome Discoverer (Thermo) was used for generating MSMS peak lists and in-house Protein Prospector, v:5.10.17 was used for database search against the *Brachypodium distachyon* sequences from the UniProtKB.2014.3.12.random.concat (30183/53249714 entries searched) database. The following search parameters were used for tryptic peptides: carbamidomethyl Cys as constant, oxidation of Met, pyro-Glu from Gln and protein N-terminal acetylation as variable modifications. Only fully specific peptides were considered with the maximum of 1 missed cleavage site. Mass tolerance was set to 5 ppm for the survey and 0.6 Da for the MSMS measurements. Minimum protein and peptide score was 51 and 20 respectively as acceptance criteria. False discovery rate was calculated for each 2D gel spot identification and found to be less than 1% in all cases. (FDR: 2*number of decoy peptide match/number of identified spectra.) In the case of homologous proteins, the one with the highest protein score and the bigger sequence coverage was listed. Three dominant proteins from each spot were selected for further *in-silico* analyses.

### Sequence Analyses and Epitope Predictions

Protein sequences identified based on the on-line nanoLC-MS/MS analysis were retrieved from the UniProt database and were used for detailed epitope mapping analyses and protein characterizations. p-BLAST was used to find protein homologues in *Poaceae*. Celiac disease-specific linear T-cell and B-cell epitopes were collected from the ProPepper database^15^. Epitope mapping was carried out using motif search algorithm of the CLC Genomic Workbench (8.5.1). A threshold of minimum 70% sequence identity was used to identify wheat epitope homologs in *Brachypodium* protein hits. A minimum epitope length of 4 amino acids was used for 100% hits, while for peptide homologs with imperfect epitope matching six consecutive amino acids were used as the limit. MHC-II peptide binding prediction algorithm of the IEDB immune epitope database and analysis resource was used to predict protein regions with possible T-cell specific linear epitopes. MHC-II alleles, DQA1*05:01/DQB1*02:01 (DQ2.5) and DQA1*03:01/DQB1*03:02 (DQ8) were used to the predictions. The predictions were made on 1/7/2016 using the IEDB analysis resource Consensus approach^16^,^17^. Using a 14aa sliding window, 15aa long peptides were produced from the analysed protein sequences. Binding affinity values of these peptides library were compared against a learning set of peptides similar in size randomly selected from the Swiss-Prot database and were used as percentile rank^18^. Peptides with small percentile ranks represented good binders. A cut-off value of top 1% was used to select potential peptides with the strongest binding abilities. Predicted T-cell binding epitopes were mapped to the selected protein sequences and also utilised in the structure analyses. Intrinsically disordered regions and binding ability of the immune reactive protein hits was analysed by the ANCHOR analysis web server^19^. Positions of epitope hits highlighted and their relative positions evaluated. Surface accessibility was determined using the Emini algorithm and Kolaskar-Tongaonkar antigenicity scale. Protein structure prediction was carried out using the I-TASSER protein structure and function prediction suite^20^. Identified B-cell and predicted T cell epitopes were highlighted on the structure using CLC Genomics Workbench (8.5.1).

## Results

**Figure 1:**
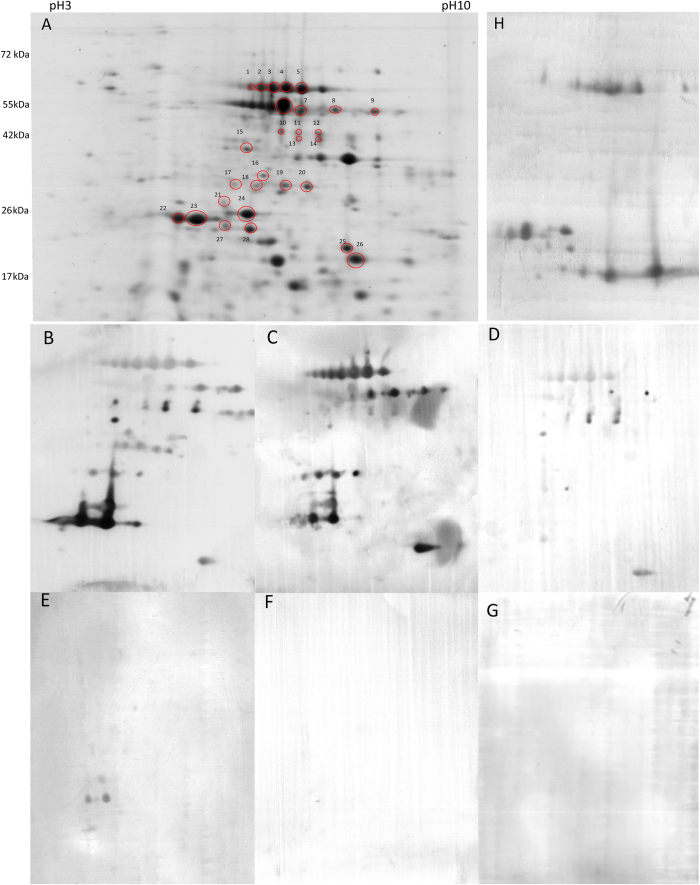
Identification of celiac disease-related proteins of *Brachyprodium distachyon* ‘Bd21′ using anti-IgA detection and patients’ blood sera. (**A**) - 2D gel electrophoresis of total protein extract of inbred line Bd21. Proteins were separated on 3–10 pH IPG strips followed by separation on 12% acrylamide gels. Labelled protein spots represent immune-reactive proteins and were sent for on-line nanoLC-MS/MS analyses. Molecular weight range is marked on the left-hand side. (**B** to **F**) - representative immunoblots using sera with IgA reactivity of therapy naïve celiac disease patients (**B** – HLA DQ2, **C** – HLA DQ8, **D** – HLA DQ8/X), ileocolic affected therapy naïve Crohn’s disease patient (**E**), HLA DQ2 celiac disease patient on gluten free diet and a healthy control (**G**). **H** –Western blot analysis of total protein extract was performed using rice glutelin antibodies^12^ and anti-rabbit IgA (Sigma-Aldrich) as secondary antibody.

*Brachypodium distachyon*, a model plant of monocot species with low prolamin content was investigated to characterise immune reactivity against non-prolamin proteins in the seed. Altogether 152 proteins spots were detected with Coomassie Brilliant Blue (CBB) staining from which 28 immune-reactive proteins spots were analysed by on-line nanoLC-MS/MS. Antibody reactivity against *Brachypodium* proteins was detected in all the celiac disease patients and two of Crohn’s disease patients, but in the latter only against two protein spots (spot 27 and 28) (Fig. 1). For detailed results see the Supplementary Table S1. While positive IgA reactions of celiac serum samples were detected against proteins from a wide range of molecular weight (approximately 15 kDa to 65 kDa) and variable isoelectric points, the protein spots showing immune reactivity with Crohn’s disease serum samples possessed an approximate molecular weight of 24 kDa. In pilot experiment for IgG reactivity and using three of the celiac serum samples, we only found a non-specific reaction with *Brachypodium* protein spots 27 and 28 (results not shown). No proteins reacted when sera of healthy controls and sera of patients on strict gluten free diet were applied. Overall experimental layout is shown in the Supplementary Fig. S1.

Predominant immune-reactive proteins are presented in Table 1 along with their protein family classification, identified homologs and number of reactive spots. Additional protein hits with their protein score, the number of unique peptides and the sequence coverage are presented in Supplementary Table S2. Most of the spots were identified as 7S or 11–12S type seed storage globulins. Variants of 7S-type storage globulins (also called Glb-1 in cereals), were mostly identified from spots within the molecular weight range of 35 to 65 kDa (spot ID 1–6, 8–15, 17–18). 11–12S storage globulins (spot ID 7, 22–28) were identified in the region of below 30 kDa. A few prolamin-type storage protein sequence hits were also identified as secondary or tertiary protein hits with similar sequence coverage values: gamma gliadin-like proteins (spot ID 1, 2, 3), HMW glutenin-like proteins – (spot ID 17–18) and LMW type glutenins (spot ID 28–29), respectively. Some enzymes and proteins with non-storage function were identified in the 35 kDa region, like glucose/ribitol dehydrogenase (spot ID 16, 18, 19, 20), aldo-keto reductase (spot ID 20), xyloglucan endo-transglucosylase/hydrolase (ID 21) and aspartic peptidase (spot ID 14) (Table 1, Supplementary Table S2). Our findings show that multiple protein spots were identified as the same protein with slightly different mass or pI values. These results are confirmed by other studies showing that post-translational modification events, such as phosphorylation and glycosylation are common phenomena in the mature *B. distachyon* seed proteome. The 7S, 11S-and 12S type globulins had occurred from 22 kDa to 64 kDa with different pI values^5^,^21^ (for details see the Supplementary Info. S1).

Two 7S globulins (Uniprot ID I1GPS5 and I1GMC8) were predominantly identified from the spots. I1GPS5 was recognised as a primary hit from 14 protein spots. This protein showed the highest similarity to the Globulin-3 and Globulin-3A wheat storage protein homologs (Uniprot ID B7U6L4 and I6QQ39). I1GMC8 was identified in two protein spots and showed the highest sequence similarity to a Globulin 1S type protein identified from *Triticum urartu* (M7ZQM3). I1HNH9 *Brachypodium* protein showed the highest homology to a 11S globulin (*A. sativa* Q38780). I1HMK7 and I1IPF2 proteins were identified as 12S globulins, with strongest similarities to 12S seed storage proteins in *A. sativa* (P12615 and P14812). Sera most frequently reacted to the Globulin-3A wheat storage protein homolog (69.23% - I1GPS5) and the 11S and 12S type globulins (76.92% - I1HNH9 and I1HMK7). A low-molecular-weight glutenin-like homolog (69.23% I1HMR6) was also highly immune reactive to the celiac disease patients’ sera.

To prove the presence of seed storage globulins within the immune reactive protein spots, Western-blot analysis was carried out using primary antibodies specific for rice glutelins, the rice homologs of 11S seed storage globulins (Fig. 1H). Strong globulin signals were detected from the protein spots ID 1 to 14 and 22 to 28.

**Figure 2:**
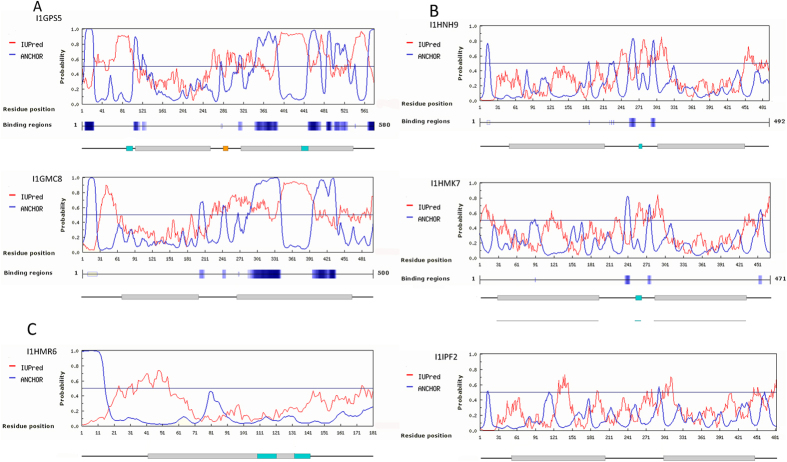
Secondary structure analyses of the identified celiac disease-related proteins. Disordered regions and disordered binding regions were predicted using ANCHOR. Red curves (IUPred) represent intrinsically disordered regions, blue curves (ANCHOR) predicts binding regions using primary sequence information. Binding regions are labelled on the sequence with dark blue. Mapped B cell epitope homologs (labelled with cyan blocks) and the identified type-I diabetes-related T-cell epitope (labelled with orange block) are presented along with the identified cupin-1 and gliadin protein domains (labelled with grey blocks). (**A**) Disordered regions were identified in 7S globulin-type proteins (I1GPS5 and I1GMC8) at the N-terminal domain and in the second cupin-1 domain. Strong binding regions were preceding both cupin-1 domains, and some binding regions were located in the second cupin-1 domain. Mapped gluten protein specific linear B-cell epitope homologs were found in proximity to some of the disordered binding regions. Type-1 diabetes-related T-cell epitope was characteristic on I1GPS5 protein and was located between the two cupin-1 domains. (**B**) Disorder was less characteristic on 11S–12S globulins (I1HNH9, I1HMK7 and I1IPF2). Two weaker binding regions were predicted between the cupin-1 domains. B cell epitope homologs were mapped in the same region. I1IPF2 protein did not contain any binding regions. (**C**) The identified prolamin-like protein (I1HRM6) contained a longer disordered section before the gliadin domain. Two B cell epitope homologs were mapped to the gliadin domain.

The most significant globulin hits (I1GPS5, I1GMC8, I1HMK7, I1IPF2 I1HNH9) as well as the most frequently identified prolamin protein (I1HRM6) were subjected to *in-silico* sequence analyses structure prediction. Two adjacent cupin-1 domains characteristic both on 7S and 11S–12S seed storage globulins were found in all of the *Brachypodium* globulin hits (Fig. 2). Regions with possible binding capabilities to structured protein partners^22^ were found in most of the analysed globulin proteins. Strong binding regions were located at the border of ordered-disordered regions in the 7S-type globulins (I1GPS5 and I1GMC8), while significantly weaker binding regions were found in the 11S and 12S globulins, and no binding region was found in the analysed *Brachypodium* prolamin sequence. (Fig. 2)

Ten amino acid long peptides with over 70% sequence homology to B-cell specific linear gluten epitopes were identified in close proximity to the binding regions of the I1GPS5 protein (Table 2). The identified epitope homologs represented peptides with polyQ stretches and were positioned in two glutamine-rich regions of the protein. Additionally, a six residues long peptide (QPEQPF) was identified in the 11S seed storage globulin, I1HNH9. This peptide was the deamidated version of a known immune reactive AGA specific B cell epitope QPQQPF (IEDB Epitope ID 147232) that gets deamidated during celiac disease process. This deamidated version represents one of the primary targets of serum DGP antibodies in celiac disease. Interestingly, none of the I1HNH9 *Poaceae* homologs contained this deamidated peptide. No epitope homologs were found in the metabolism-related proteins.

**Figure 3:**
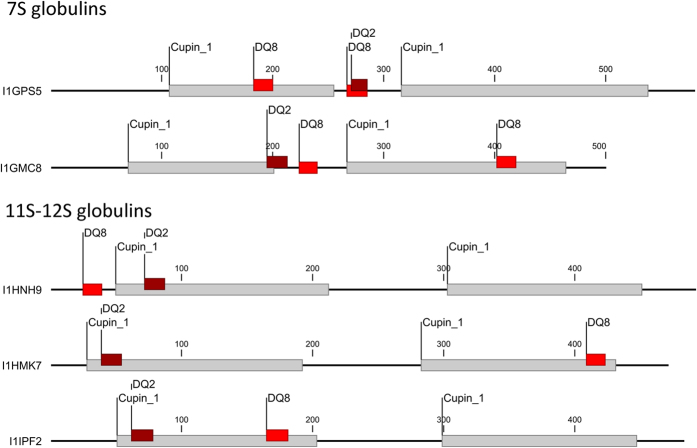
MHC II class T cell epitope prediction of *Brachypodium* 7S and 11S–12S globulin proteins using HLA DQ2 and DQ8alleles and IEDB analysis resource Consensus tool. Selection of predicted binders was carried out using top 1% binders based on consensus percentile rank values. Predictions were calculated for each allele separately. Predicted epitopes are mapped to the protein sequences.

When gluten related known T-cell epitopes were mapped to the *Brachypodium* proteins, no known epitopes were found. However, a type-I diabetes-specific T-cell epitope, EEQLRELRRQ^23^ was identified from I1GPS5 with 100% sequence identity at the position 281. To check for novel T cell epitopes, MHC-II binding predictions were carried out from the main 7S, and 11S–12S globulin hits using HLA-DR3-DQ2 and HLA-DR4-DQ8 MHC-II haplotypes (Fig. 3 and Supplementary Fig. S2). DRB1*0301, DRB1*0405 DRB1*0401 and DRB1*0402 HLA alleles were used as primary type-I diabetes-related MHC-II alleles (Supplementary Fig. S2). In the analysed 7S globulin protein (I1GPS5) predicted DQ2 and one of the predicted DQ8 epitopes were located between the two cupin-1 domains (the I1GPS5 section between residues 254 to 314). This region also harboured the published T1D epitope in the 281–290 aa region (Supplementary Fig. S2). Some of these potential epitope regions and the core region of the DQ2 and DQ8 epitopes were positioned at the surface of the protein molecule. There were epitopes also overlapping with peptides remaining intact after *in-silico* digestion of the protein sequences using gastrointestinal enzymes, such as pepsin, trypsin or chymotrypsin, respectively (Supplementary Fig. S3). Further DQ8, DR3 and DR4 specific peptide candidates were found in the first cupin-1 domain. Contrary, no epitope-dense region was predicted between the two cupin-1 domains in the 11S and 12S seed storage globulins (I1HNH9, I1HMK7 and I1IPF2) (Fig. 3 and Supplementary Fig. S2).

**Figure 4:**
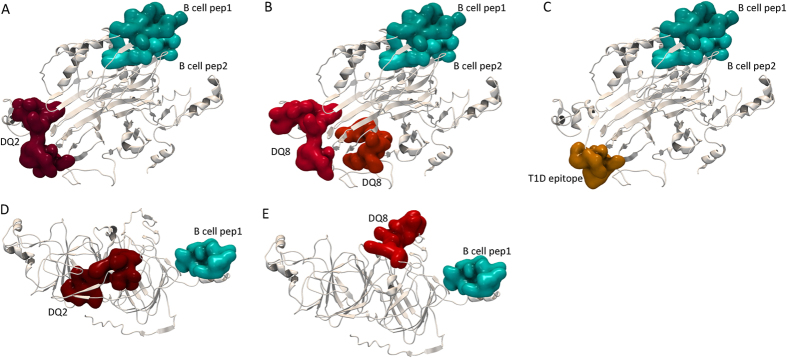
*In-silico* structure prediction of the *Brachypodium* 7S globulin (I1GPS5) and 11S globulin (I1HNH9) proteins. Monomer globulin structures were modelled using publicly available plant orthologues as structural templates and the I-Tasser protein structure and function prediction server. Positions of identified gluten specific B cell epitope homologs and the predicted HLA DQ2 and DQ8 specific T cell epitopes were mapped to the structure. (**A**–**C**) I1GPS5; (**D**,**E**) I1HNH9.

Tertiary structure model of the I1GPS5 7S globulin monomer was built using publicly available crystal structures of 7S globulins isolated from plants. The identified gluten specific B cell epitope homologs that are in about 350 amino acid distance from each other in the sequence were mapped in close structural proximity. One of the B cell epitope homologs is located on the outer surface of the beta-barrel formed by the second cupin-1 domain. The predicted HLA-DQ specific T-cell epitopes and the identified type-I diabetes epitope were located at the opposite site of the protein molecule also potentially exposed to digestive enzymes (Fig. 4A,B,C). In the case of the 11S seed storage globulin I1HNH9 the predicted DQ2 epitope was in a buried position while the B cell epitope homolog QPEQPF located on the surface of the protein (Fig. 4D and E).

## Discussion

Based on our previous studies a high number cereal grain proteins may contain peptides rich in proline and glutamine making them a target for celiac disease-related antigen presenting cells and immunoglobulins^10^,^15^,^24^. In wheat, the majority of these proteins belong to the prolamin superfamily and are a natural compound of the wheat gluten. However, there are some non-prolamin type proteins that also contain peptides identical or strongly similar to gluten-specific T-cell and B-cell epitopes^10^,^15^.

Some previous studies have focused on the investigation of immune reactivity and toxic behaviour of non-gluten proteins of wheat related to celiac disease^25^,^26^,^27^,^28^. Recently, Huebener and colleagues have analysed the possible involvement of non-gluten proteins as target antigens in celiac disease related humoral response^29^. Serine-protease inhibitors, alpha-amylase inhibitors, farinins and seed globulins have demonstrated a significant immune response. Farinins, alpha-amylase inhibitors and serine–protease inhibitors are proteins with a conserved cysteine skeleton highly similar to that of prolamins and without the presence of long Q and P rich repetitive regions^30^. Additionally, 35% of celiac disease patient’s sera showed reactivity against protein spots identified as seed globulins using the protein extract of ‘Butte86′ wheat^28^. Increased celiac serum antibody reaction was also measured against cereal globulin extracts by Troncone *et al*^27^. Seed storage globulins, like Glo-3A from wheat, were related to celiac disease and type-I diabetes in a high-risk type-I diabetes population^31^,^32^,^33^. Celiac disease cases having first-degree type-I diabetes relatives showed a higher response to Glo-3A protein at the early stage of illness even prior transglutaminase-2 autoantibodies were detected^32^. They suggested that Glo-3 wheat globulins are either antigenic and participate in the development of these diseases or the high immune reactivity against these proteins indicates an abnormal gut barrier function, lack of oral tolerance or cross-reactivity with self-antigens. Epitope patterns of I1GPS5 and wheat Glo-3A (B7U6L4) along with some other orthologues found in wheat and related cereal species highlighted epitope dense conserved binding regions between the two cupin-1 domains along with some other conserved positions in both cupin-1 domains (Supplementary Fig. S4).

We also investigated whether increased unspecific peptide recognition due to the increased gut permeability in Crohn’s disease patients would cause antibody reactivity. The lack of immune response with Crohn’s disease sera and sera from celiac patients on a strict gluten-free diet strengthen the observations that these reactions were specific for the active celiac disease. Our results thus indicate that in celiac disease, not only prolamins but also non-prolamin storage proteins, like the *Triticum* homologues of 7S and 11S–12S globulins can trigger B cell activation and humoral response with potentially adverse effect on the villi. Sequence homologs with polyQ chains or natural deamidated variants of immune reactive peptides like the B cell peptide in the 11S seed storage globulin I1HNH9 can also serve as antigens. The sequence of a known highly immunogenic epitope QPQQPF is present in a natural deamidated version (QPEQPF) in one of the 11S seed storage globulins. The PQQ to PEQ modification has a proven positive effect on the strength of antibody binding^34^. Although 7S and 11S–12S seed storage globulins both represent strongly conserved protein families with cupin-1 domains in cereals our epitope analyses highlighted some remarkable differences between the protein families (Supplementary Fig. S2). These differences indicate the presence of possible sub-classes with various immune reactive potential. The amount of these unique globulin sub-classes can also be different in the grains of the different species, with a significantly lower amount expressed in wheat and in cereals, where prolamins serve as major storage protein components. This fact partially explains why these proteins were overlooked compared to the most abundant prolamins in wheat, rye or barley. Grains of cereals as oat, rice or *Brachypodium distachyon* are enriched in seed storage globulins.

Our study confirmed that globulin-type cereal seed storage proteins are specifically related to celiac disease, as patients suffering in other immunological, inflammatory diseases, like Crohn’s disease did not recognise these globulin-type cereal storage proteins. Adverse results of the immunoblot analyses with sera of celiac patients on a gluten-free diet had also strengthened the assumption that seed storage globulins may act as secondary B cell stimulants due their strong sequence homology to epitopes originated from the primary gluten triggers. In progressive stages of the disease the villous atrophy and the increased gut permeability contributes why these proteins can serve as cross antigens. The recovered intestinal mucosa of the celiac patient on a strict gluten-free diet prevents better the passage of ingested proteins and probably, in this way can be the strong immune reactivity controlled.

On the other hand, prediction novel HLA class II restricted T cell epitopes with binding ability to HLA DQ2 and DQ8 molecules raise the possibility that these protein families may also be involved in the development of the disease. The conserved presence of predicted HLA DQ2 and DQ8 binding regions that also overlap with known and predicted type-1 diabetes-related epitope regions implicate that these proteins can also serve as trigger molecules in these diseases. Type-I diabetes and celiac disease share a similar genetic background, as both conditions are associated with the presence of HLA DQ2 and HLA DQ8 haplotypes. This common genetic background can partially explain the relatively high prevalence of patients with both diseases. In patients diagnosed with both diseases, type-I diabetes develops first, suggesting that strict gluten-free diet may decrease the risk of diabetes if implemented in the prediabetic period^35^. Interestingly, none of the patients involved in our study was diagnosed with type-I diabetes at the stage when celiac disease was diagnosed. However, one patient with an MHC II haplotype HLA DQ8 neglecting the gluten free diet was also diagnosed with type-I diabetes several years later. Studies have revealed about ten times higher risk of development of celiac disease in children with type I diabetes^36^. Studies focusing on a population-based screening of celiac disease with subsequent type-I diabetes^37^,^38^ have revealed an increase in type 1 diabetes before the age of 20 years in individuals with prior celiac disease. The age distribution of celiac patients involved in our study varied from 1.4 years to 13.5 years, all of them under 20 years. Although the prevalence of type-I diabetes developed later is not known for these patients, except for one case, when gluten free diet was neglected, based on previous findings, ratio of celiac patients with subsequent diagnoses of type-I diabetes is rather low. In the study of Ludvigsson *et al*.^38^ only ca. 1.04% of individuals with celiac disease were diagnosed with type-I diabetes in the later stage. Shared nutritional factors, such as presence of gluten-containing cereals in the daily diet and common HLA DQ2 and DQ8 haplotypes may explain this combined risk.

## Conclusions

In summary, our results indicate that both in celiac disease and type-I diabetes MHC-II presentation and B cell response may be developed not only for prolamins but also for seed storage globulins even in distant relatives of wheat, such as *Brachypodium distachyon,* having seed storage globulins similar to oat and rice. *Brachypodium distachyon* accession Bd21 with a small diploid genome size (272 Mbp) offers many advantages, such as self-fertility, simple nutrient requirements and short lifecycle. Sequencing and annotation of the Bd21 genome were recently completed, making further functional proteomic studies feasible. All these features make the *Brachypodium distachyon* an excellent research material, even though it is at present not directly considered for human use. However, its seed storage proteins, especially globulins, belong to quite conserved proteins in plants, which when eaten, may cause some problems due to the presence of some B-cell epitope homologs and possible T-cell reactive peptides present in the globulin fraction. Therefore, such cereals would not be harmless food alternatives for celiac patients despite negative results on conventional R5 ELISA for celiac-related toxicity (data not shown). High-resolution 2D gel electrophoresis followed by immunoblotting and protein identification have proved that 7S and 11S–12S seed storage globulins may act as antigens for celiac disease specific IgA antibodies. Structure prediction analyses and epitope mapping results indicate that these proteins do not contain known gluten related T cell epitopes. However, additional epitopes predicted with strong binding capabilities to HLA DQ2 and HLA DQ8 are present between the two cupin-1 domains or at the surface of the beta-barrel formed by the domain of the cereal 7S and 11S–12S globulins. Storage globulins are only present as contaminants in the wheat gluten; therefore they play a less significant role. Contrary to this, seed storage globulins are the main source of nutrient storage in cereals like rice, oat or *Brachypodium*^5^. Their presence in the endosperm of these cereal species is more remarkable compared to that in wheat or barley. Despite the strongly conserved structure of 7S globulins, proteins like Glo3A in wheat and I1GPS5 in *Brachypodium* and some of the 11S–12S globulins represent a special class of seed globulins with an epitope dense region between the two cupin-1 domains and therefore might represent a higher risk for celiac disease patients. Our study draws attention on the presence of conserved seed storage protein families in various cereal species, such as wheat, oat, rice and *Brachypodium*. Although 7S and 11S–12S seed globulins are present in low amount in the wheat grain, they represent major storage protein groups in species like oat or rice. Therefore the presence of some epitopes in highly conserved regions may be also characteristic on orthologues in other species. Mapped and predicted epitopes found in these proteins suggest that both in celiac disease and type-I diabetes MHC-II presentation and B cell response may be developed not only for prolamins but also for seed storage globulins. The level of the response to globulins may depend on the type of seed globulins and amount of proteins with immune responsive peptide content. It is also suggested to perform further investigations whether diets enriched in seed storage globulins (like rice or oat) inhibit the sufficient healing, especially in patients with combined high risk to type-I diabetes and proven susceptibility to these proteins.

## Additional Information

**How to cite this article:** Gell, G. *et al*. Characterization of globulin storage proteins of a low prolamin cereal species in relation to celiac disease. *Sci. Rep.* **7**, 39876; doi: 10.1038/srep39876 (2017).

**Publisher's note:** Springer Nature remains neutral with regard to jurisdictional claims in published maps and institutional affiliations.

## Supplementary Material

Supplementary Figures

Supplementary Table S1

Supplementary Table S2

## Figures and Tables

**Table 1:** Identified *Brachypodium distachyon* protein hits, and their immune reactivity against serum IgA antibodies originating from celiac disease patients, Crohn’s disease patients, healthy controls and celiac patients on gluten free diet.

Brachypodium hit (Uniprot ID) identified by on-line nanoLC-MS/MS	Protein family type	Best *Poaceae* homolog	Number of reactive protein spots on 2D GE	Number of celiac disease sera with IgA reactivity (n = 13)	Number of Crohn’s disease sera with IgA reactivity (n = 10)	Number of GFD celiac sera with IgA reactivity (n = 3)	Number of healthy controls with IgA reactivity (n = 8)
I1GPS5	7S globulin	Globulin 3 (B7U6L4, *T. aestivum*)	14	9	0	0	0
I1GMC8	7S globulin	Globulin 1S (M7ZQM3, *T. urartu*)	2	8	0	0	0
I1HNH9	11–12S globulin	11S globulin (Q38780, A sativa)	2	10	2	0	0
I1HMK7	11–12S globulin	12S seed storage globulin (P12615, A sativa)	4	10	2	0	0
I1IPF2	11–12S globulin	12S seed storage globulin (P14812, A sativa)	1	8	0	0	0
I1HMR6	Prolamin–like	LMW-GS (H6VLQ5, *T. aestivum*)	1	9	0	0	0
I1HIH2	Metabolism related proteins	Aldose reductase (M7Z0 × 1, T urartu)	1	8	0	0	0
I1HM37	Metabolism related proteins	Glucose and ribitol dehydrogenase-like protein (M8C904, Ae tauschii)	1	5	0	0	0
I1H5 × 3	Metabolism related proteins	Uncharacterized protein (M8BEB8, Ae. tauschii)	1	5	0	0	0
I1GNN1	Metabolism related proteins	Xyloglucan endotransglucosylase/hydrolase (Q56TP4, T aestivum)	1	4	0	0	0

Numbers of immune reactive sera represent patients reacting on protein spots within a particular protein type.

**Table 2:** Gluten specific B cell epitope homologs of the identified *Brachypodium distachyon* proteins.

Protein	Homologue region and position	Celiac disease-specific B-cell Epitope Sequence	Matched epitope IEDB ID	Sequence homology
I1GPS5	DEKQQQQQQQESR_89–101_,	LQQQQQQQQQ, QQQQQQQQQI QKQQLQQQQQ QQQQQQQQLQ QQQQQQQPLS QQQQQQQILQ	148680 148881 148771 148880 149441 148877	70%
I1GPS5	QGRGQQQQQQQGRY_436–449_	LQQQQQQQQQ, QQQQQQQQQI QQQQQQQQLQ QQQQQQQPLS QQQQQQQILQ QPISQQQQQQ	148680 148881 148880 149441 148877 149432	70%
I1GMC8	—	—	—	—
I1HNH9	QPEQPF_271–276_	QPQQPF	147232	83%
I1HMK7	QKQPFLPIEP_252–261_	QPQPFLPQQP	149434	70%
I1IPF2	—	—	—	—
I1HMR6	CQQLALIPVQSR_110–121_	CQQLWQIPEQ QLWQIPEQSR	149280 149429	70% 70%
I1HMR6	FKQQLGQGQQ_133–142_	QKQQLQQQQQ	148771	70%
I1HIH2	—	—	—	—
I1HM37	—	—	—	—
I1H5 × 3	—	—	—	—
I1GNN1	—	—	—	—

The sequence of homologue region and position in the protein. The mapped epitopes and their IEDB epitope IDs are highlighted. Sequence homology based on which the peptide was accepted as an epitope homologue is described in the materials and methods. The QPEQPF sequence is identical to the processed (deamidated) form of the corresponding wheat epitope.
