# Supplementary material for: Characterization of globulin storage proteins of a low prolamin cereal species in relation to celiac disease
Source: Sci Rep. 2017 Jan 4;7:39876. doi: 10.1038/srep39876 (PMC5209737; doi:10.1038/srep39876)
Supplement: Supplementary Figures [file srep39876-s1.pdf]

Supplementary Material

Characterization of globulin storage proteins of a low prolamin cereal species in relation to celiac disease

Gyöngyvér Gell[1¶], Krisztina Kovács[1¶], Gábor Veres[2], Ilma R. Korponay-Szabó[3] and Angéla Juhász[1*†]

[1]Agricultural Institute, MTA Centre for Agricultural Research, Department of Applied Genomics Martonvásár, HU 2462, Hungary
[2]Semmelweis University of Medicine, 1st Department of Pediatrics, Budapest, HU 1083, Hungary
[3]Coeliac Disease Center, Heim Pál Children's Hospital, Budapest, HU 1089 and Department of Pediatrics, Clinical Center, University of Debrecen, Debrecen, Hungary
[†] Australia-China Centre for Wheat Improvement, School of Veterinary and Life Sciences, Murdoch University, Murdoch, 6150, Western Australia

Supplemental file 1	Figure S1	Overall Overall experimental layout of this study.
Supplemental file 2	Figure S2	MHC II class T cell epitope prediction of *Brachypodium* 7S and 11S-12S globulin proteins.
Supplemental file 3	Figure S3	Peptides resistant to gastrointestinal enzymes and protein surface accessibility and flexibility characteristics.
Supplemental file 4	Figure S4	Orthologs of I1GPS5 7S globulin and their predicted HLA DQ2, DQ8, DR3 and DR4 epitopes identified from Triticeae.
Supplemental file 5	Info S1	Post-translational protein modifications in *Brachypodium distachyon*.
Supplemental file 6	Table S1	Immune response of each analysed blood serum sample.
Supplemental file 7	Table S2	List of identified protein hits are presented for each spot separately.

Supplemental figure 1

Overall experimental layout of this study

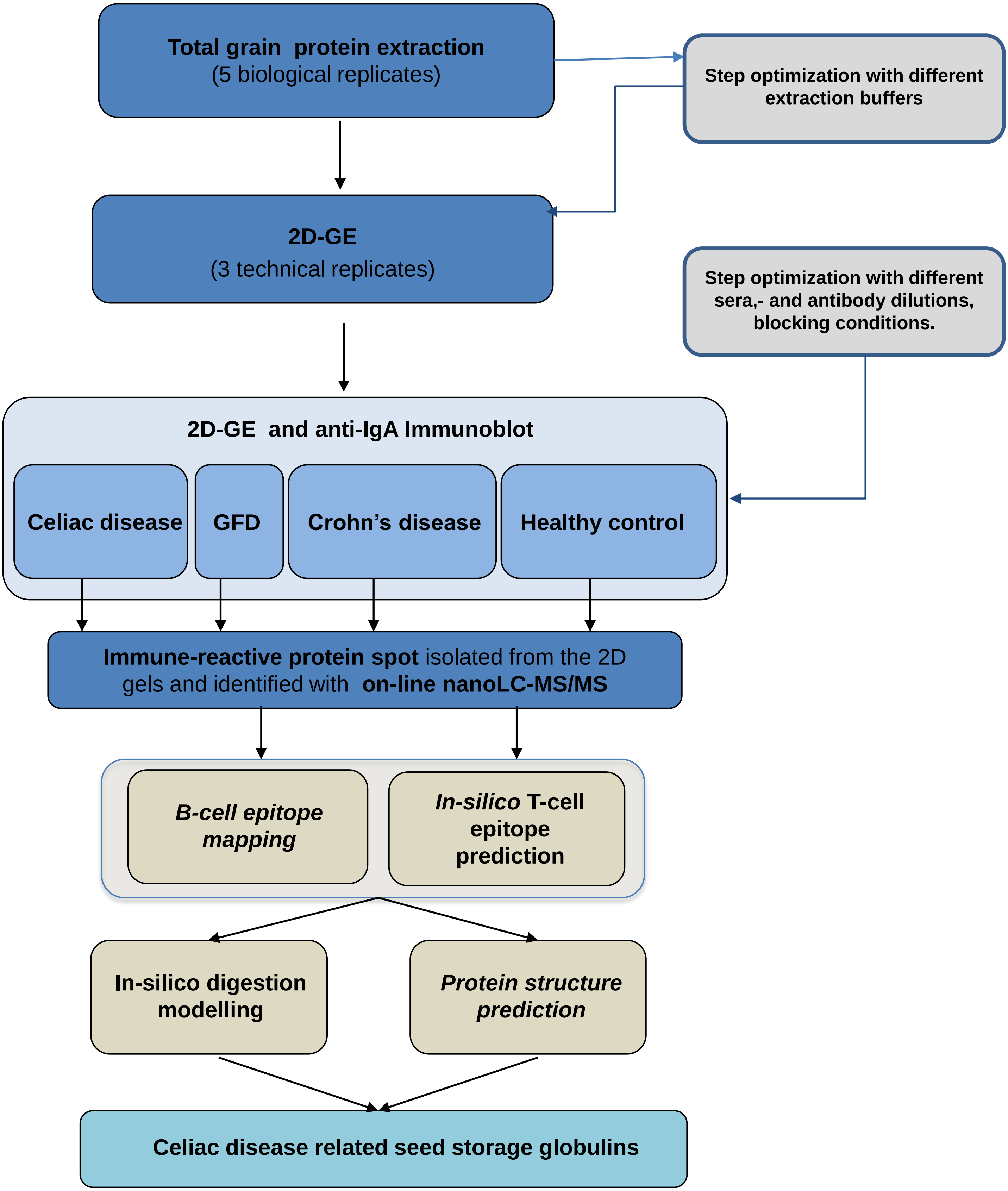

Supplemental figure 2

MHC II class T cell epitope prediction of Brachypodium 7S and 11S-12S globulin proteins using HLA DQ2, DQ8, DR3, DR4.5, DR4.1 and DR 4.2 alleles and IEDB analysis resource Consensus tool.
Selection of predicted binders was carried out using top 1% binders based on consensus percentile rank values. Predictions were calculated for each allele separately. Predicted epitopes are mapped to the protein sequences. Predicted epitope profile of I1GPS5 7S globulin sequence show highly similar epitope distribution to the Glo-3A wheat homolog in conserved positions.

7S globulins

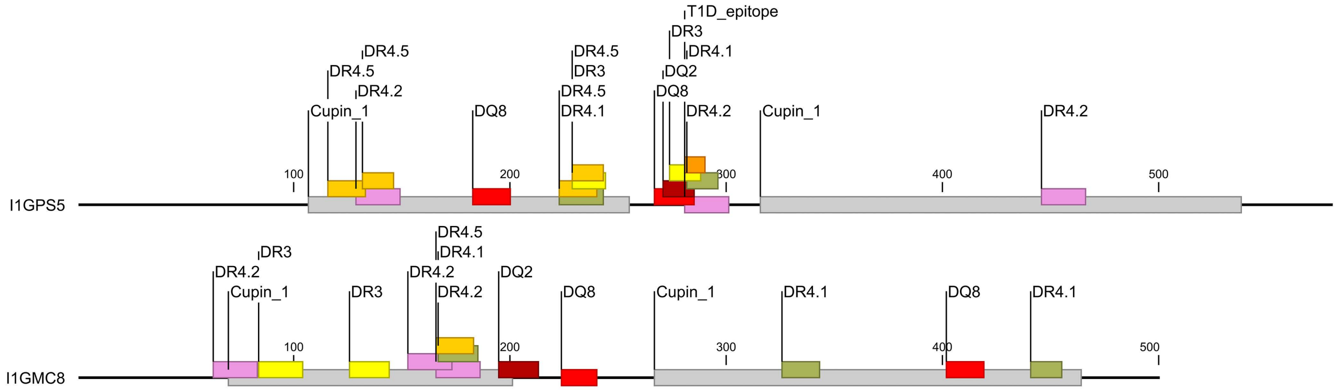

11S-12S globulins

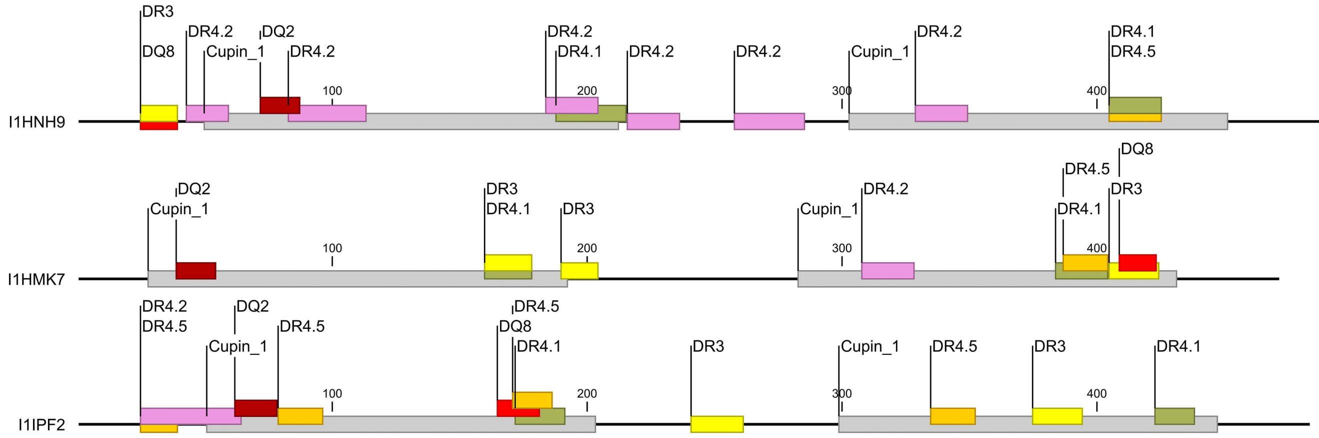

Supplemental figure 3

Peptides resistant to gastrointestinal enzymes and protein surface accessibility and flexibility characteristics
Intact peptides of 7S globulin I1GPS5 and 11S globulin I1HNH9 generated by in-silico digestion of the protein sequences using consecutive digestion with pepsin (pH<2), trypsin and chymotrypsin (low specificity). Digestion patterns were made using the PDMQ method (1). Light blue rectangles label peptide regions intact after digestion with pepsin. Middle blue label represents peptides resistant to digestion both with pepsin followed by trypsin. Dark blue regions represent intact peptides after consecutive digestion with pepsin, trypsin and chymotrypsin. Surface accessibility and flexibility information was calculated using the Kolaskar –Tongaonkar semi-empirical method and the surface probability method by Emini (2, 3).

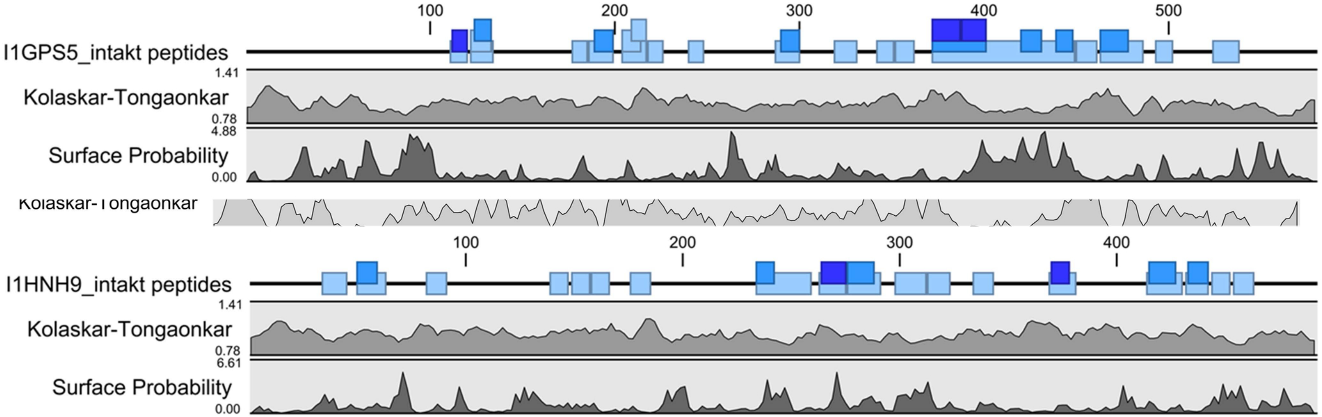

Supplemental figure 4

Orthologs of I1GPS5 7S globulin and their predicted HLA DQ2, DQ8, DR3 and DR4 epitopes identified from Triticeae. Selection of predicted binders was carried out as described before.

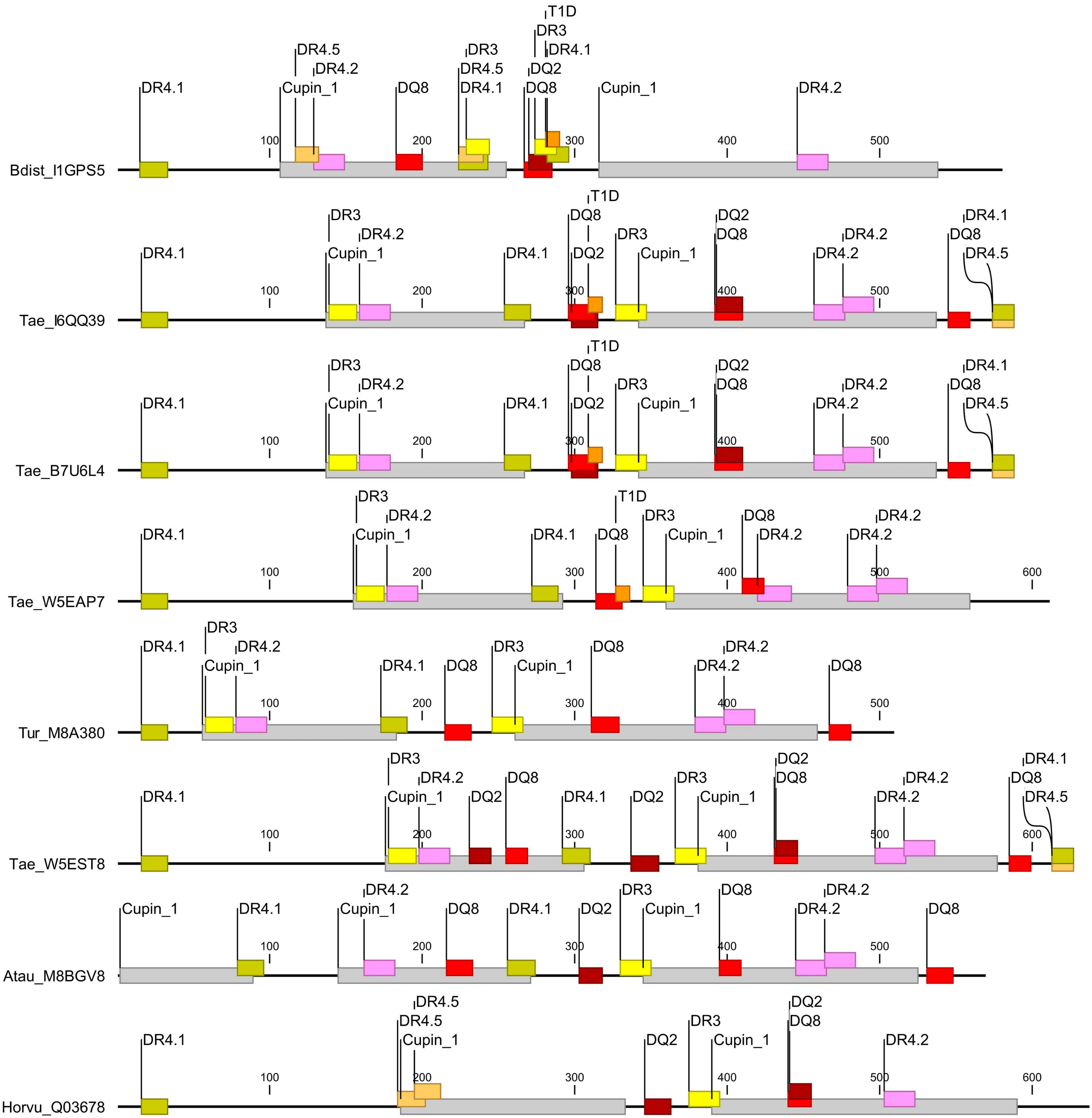

Supplementary Info S1

Post-translational protein modifications in *Brachypodium distachyon*.

The presence of the post-translational modifications, such as phosphorylations and glycosylations are common phenomena in B. *distachyon* seed proteome[4]. Seed globulins the major seed storage proteins found in *Brachypodium distachyon*, can be divided into two main classes based on their sedimentation coefficient; 7S (Vicilin-type) and 11- and 12S (Legumin-type) globulins. 7S globulins are stored in protein bodies in cereals as trimers consisting of morphological subunits of 50-70 kDa[5,6]. 12S globulins located in the starchy endosperm, being hexameric comprising morphological subunits of 60-80 kDa, that cleaved post-translationally into acidic and basic polypeptide chains linked with a disulfide bond [7,8]. These studies confirmed our findings that lots of spots were identified as the same proteins with similar but different mass or pI values. Larré and co-workers[9] made a high resolution seed proteome analyses from Osborne fractionated mature seed proteins of *Brachypodium distachyon*, and they found that the 7S and also the 11 and 12S type seed storage globulins occurred in various molecular weight and isoelectric point. In parallel with this Wang and co-workers investigated glutenin, albumin and globulin components of 13 *B. distachyon* accessions with two-dimensional gel electrophoresis (2-DE) and matrix-assisted laser desorption ionization time of flight mass spectrometry (MALDI-TOF-MS) followed by peptide massfinger printing (PMF) and S/MS protein identification. Based on the MS/MS analyses from ten different spots 7S, 11- and 12S globulins were identified[4].

1. Haraszi, R., Tasi, Sc., Juhasz, A., Makai, Sz. PDMQ - Protein Digestion Multi Query software tool to perform in silico digestion of protein/peptide sequences. BiorXiv, doi: http://dx.doi.org/10.1101/014019, (2015).
2. Kolaskar, A. S. and Tongaonkar, P. C. A semi-empirical method for prediction of antigenic determinants on protein antigens. *FEBS Lett.* **10,** 276(1-2) 172-4 (1990).
3. Emini, E. A., Hughes, J. V., Perlow, D. S., Boger, J. Induction of hepatitis a virus-neutralizing antibody by a virus-specific synthetic peptide. *J. Virol.* ***55***(3), 836-839 (1985).
4. Wang, K. et al. Characterization of seed proteome in Brachypodium distachyon. J. Cereal Sci. 52 177-186 (2010).
5. Burgess, S.R. and Shewry, P.R. Identification of Homologous Globulins from Embryos of Wheat, Barley, Rye and Oats. *J. Exp. Bot.* **37**, 1863-1871 (1986).
6. Kriz, A. L., Wallace, N. H. Characterization of the maize Globulin-2 gene and analysis of two null alleles. *Biochem. Genet.* **29**(5-6):241-254 (1991).
7. Shotwell, M. A. Oat globulins. Seed proteins (Eds. P. Shewry and R. Casey), 389-400, (Kluwer Academic Publishers, 1999).
8. Takaiwa, F., Ogawa, M., Okita, T.W. Rice glutelins. Seed proteins (Eds. P. Shewry and R. Casey), 401-425, (Kluwer Academic Publishers, 1999).
9. Larré, C. et al. *Brachypodium distachyon* grain: identification and subcellular localization of storage proteins. *J. Exp. Bot.* **61**(6), 1771–1783 (2010).
